# Endoscopic Combined Intrarenal Surgery Versus Percutaneous Nephrolithotomy for Complex Renal Stones: A Systematic Review and Meta-Analysis

**DOI:** 10.3390/jpm12040532

**Published:** 2022-03-28

**Authors:** Yung-Hao Liu, Hong-Jie Jhou, Meng-Han Chou, Sheng-Tang Wu, Tai-Lung Cha, Dah-Shyong Yu, Guang-Huan Sun, Po-Huang Chen, En Meng

**Affiliations:** 1 Department of Surgery, Division of Urology, Tri-Service General Hospital, National Defense Medical Center, Taipei 114, Taiwan; michael40315@yahoo.com.tw (Y.-H.L.); princecharmingben@gmail.com (M.-H.C.); doc20283@gmail.com (S.-T.W.); tailungcha@gmail.com (T.-L.C.); yuds45@gmail.com (D.-S.Y.); ghsun@gmail.com (G.-H.S.); 2 Department of Neurology, Changhua Christian Hospital, Changhua 500, Taiwan; xsai4295@gmail.com; 3 Department of Internal Medicine, Tri-Service General Hospital, National Defense Medical Center, Taipei 114, Taiwan

**Keywords:** endoscopic combined intrarenal surgery, percutaneous nephrolithotomy, complex renal stones, stone-free rates, safety, efficiency

## Abstract

Background: Endoscopic combined intrarenal surgery (ECIRS) adds ureteroscopic vision to percutaneous nephrolithotomy (PCNL), which can be helpful when dealing with complex renal stones. Yet, there is still no consensus on the superiority of ECIRS. We aimed to critically analyze the available evidence of studies comparing efficacy, safety, bleeding risk, and efficiency of ECIRS and PCNL. Methods: We searched for studies comparing efficacy (initial and final stone-free rate), safety (postoperative fever, overall and severe complications), efficiency (operative time and hospital stay) and bleeding risk between ECIRS and PCNL. Meta-analysis was performed. Results: Seven studies (919 patients) were identified. ECIRS provided a significantly higher initial stone-free rate, higher final stone-free rate, lower overall complications, lower severe complications, and lower rate of requiring blood transfusion. There was no difference between the two groups in terms of postoperative fever, hemoglobin drop, operative time, and hospital stay. In the subgroup analysis, both minimally invasive and conventional ECIRS were associated with a higher stone-free rate and lower complication outcomes. Conclusions: When treating complex renal stones, ECIRS has a better stone-free rate, fewer complications, and requires fewer blood transfusions compared with PCNL. Subgroups either with minimally invasive or conventional intervention showed a consistent trend.

## 1. Introduction

Renal stone is a common disorder, and complex renal stones are defined as having multiple stones or having anatomical or functional abnormalities, regardless of being peripheral or branched stones. Staghorn stones with their branching characteristics, occupying the renal pelvis and one or more calices, are the most complicated type. They usually have large stone burdens, determined by the number, diameter, and location of stones evaluated on images [1]. Since its development in 1976, percutaneous nephrolithotomy (PCNL) has been the indicated treatment for these cases with stone-free rates (SFRs) of 98.5% and 71% for partial and complete staghorn stones, respectively [2].

However, in cases with greater stone burden, PCNL is not the only option. In 1992, Dr. JG Ibarluzea utilized the clear visual field of the ureteroscope to remove stone fragments through an Amplatz sheath while performing PCNL simultaneously [3]. Later in 2008, Dr. CM Scoffone coined the term endoscopic combined intrarenal surgery (ECIRS) and operated under the Galdakao-modified supine Valdivia (GMSV) position, an adaption of the prone position [4].

ECIRS aimed to improve the one-step resolution of urolithiasis while reducing the number of access tracts [5]. Multiple retrospective studies comparing ECIRS and PCNL have reported contradictory outcomes. There is still no consensus on the superiority of ECIRS in terms of operative time, hospital stay, and even stone free rate or complications.

Furthermore, as techniques for miniaturized access in urolithiasis evolved, the mini-percutaneous access system (14–20 Fr sheath size) has been widely adopted. We have also conducted subgroup analysis for patients who underwent conventional-PCNL (cPCNL) or mini-PCNL (mPCNL) to compare the two procedures. This meta-analysis aims to compare the efficacy, safety, and efficiency between ECIRS and PCNL in patients with complex renal stones in order to provide recommendations for physicians in clinical practice.

## 2. Materials and Methods

### 2.1. Study Design

The study follows the Preferred Reporting Items for Systematic Review and Meta-analysis (PRISMA) and the Meta-analysis of Observational Studies in Epidemiology (MOOSE) [6,7] statement (Appendix A and Appendix B). The study is also registered in the Open Science Framework (OSF, DOI: 10.17605/OSF.IO/DRBFZ).

### 2.2. Search Strategy

From the inception through June 2021, databases including the Cochrane Library, PubMed, and Embase were searched. We conducted the search using subject headings and search field tags of the title, abstract, and keywords, comprised of “endoscopic combined intrarenal surgery” and “percutaneous nephrolithotomy” (details in Appendix C).

### 2.3. Eligibility Criteria

Studies that met all the following inclusion criteria were selected:

(1) Types of participants: patients with complex renal stone.
(2) Types of interventions: Studies comparing ECIRS and PCNL were eligible.
(3) Types of outcome measures: Our outcomes of interest are categorized into “efficacy”, “safety”, and “efficiency”. Studies that reported at least an outcome of interest (i.e., initial stone free rate) were included.

Moreover, the studies should provide adequate information to calculate the effect estimated for meta-analysis. We did not exclude studies based on publication date, language, or geographical area. The exclusion criteria were as follows: overlapping or duplicate publication; studies in which necessary data could not be extracted; reviews, letters, and case reports.

### 2.4. Risk of Bias Assessment

The quality of the RCTs was appraised using the Cochrane Handbook for Systematic Reviews of Interventions, Risk of Bias Tool [8]. We also used the Newcastle–Ottawa Scale for the quality of prospective non-randomized studies [9] (Appendix D).

### 2.5. Data Extraction and Outcome Measurement

Two reviewers (Y.-H. Liu and P.-H. Chen) independently extracted datasets from the eligible studies. There were nine outcomes in the current study defined as follows:

#### 2.5.1. Efficacy Outcomes

(1) Initial stone-free rate (Initial SFR): Absence of stone or residual stone fragments on plain abdominal X-ray (Kidney–Ureter–Bladder, KUB) or non-contrasted abdominal computed tomography (NCCT) within 4 weeks post-operation, or as defined by each study.
(2) Final stone-free rate (Final SFR): The stone-free status was defined as above, but was assessed after the auxiliary procedure (i.e., shock wave lithotripsy, PCNL or ureteroscopic lithotripsy).

#### 2.5.2. Safety Outcomes

(1) Overall complications: Perioperative complications were graded according to the Clavien classification system. Overall complications included all grades.
(2) Severe complications: Clavien–Dindo classification system ≥grade 2.
(3) Postoperative fever: Transient body temperature taken >38.5 °C after operation.

#### 2.5.3. Bleeding Risk

(1) Hemoglobin drop: The postoperative hemoglobin level decreased comparing with that of pre-operative evaluation.
(2) Required blood transfusion: Blood transfusion needed due to significant hemorrhage.

#### 2.5.4. Efficiency outcomes

(1) Operative time: Time taken on the operating table, from positioning to the end of the procedure.
(2) Hospital stay: Number of days since admission for pre-operative evaluation, operation, imaging for SFR assessment, and the treatment if complications occurred.

### 2.6. Quality Assessment

The Grading of Recommendations Assessment, Development, and Evaluation (GRADE) methodology was used to assess the certainty of evidence from the included studies (Appendix E) [10].

### 2.7. Subgroup Analysis, Meta-Regression, and Sensitivity Analysis

A priori subgroup analysis explored the influence of miniaturized access or conventional access of the operation on the pooled effect estimates (Appendix F). We performed a mixed-effects meta-regression analysis to evaluate the potential influence of publication date and Amplatz sheath size on the heterogeneity for the outcomes. We assessed the robustness of treatment effects on outcomes via a sensitivity analysis [11] that excluded high-risk-of-bias cohort studies (Appendix H).

### 2.8. Statistical Analysis

We analyzed dichotomous variables by calculating odds ratios (ORs) with 95% confidence intervals (CIs). The continuous variables were estimated with the mean difference (MD). Both dichotomous and continuous outcomes were calculated using the inverse variance method. We reported both random-effects meta-analysis models with the DerSimonian–Laird estimator and fixed-effect model. Statistical heterogeneity was assessed using Cochran’s Q statistic and quantified by the I^2^ statistic [12].

Publication bias was evaluated using funnel plots and Egger’s test. (Appendix I) [13] All statistical analyses were performed using the “metaphor” and “meta” [14,15] packages of R software version 4.1.0.

To obtain conclusive results [16], trial sequential analysis (TSA) was applied to calculate the diversity-adjusted required information size (RIS) and trial sequential monitoring boundaries (Appendix J). The models for all outcomes were based on an alpha of 5% and a power of 80%. TSA was performed using TSA software version 0.9.5.10 Beta (Copenhagen Trial Unit, Copenhagen, Denmark).

## 3. Results

### 3.1. Study Identification and Selection

**Figure 1:**
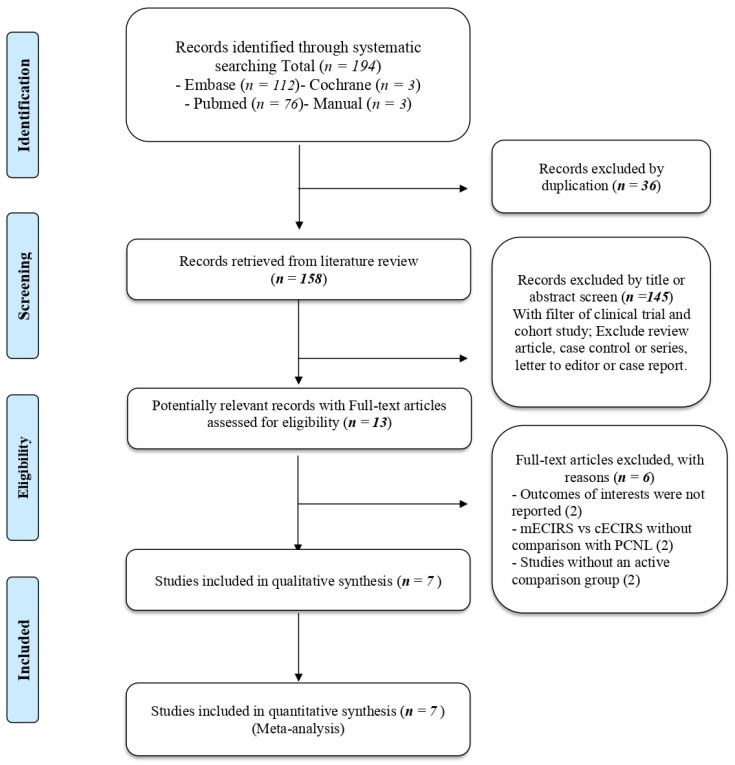
PRISMA flow diagram. mECIRS: mini-endoscopic combined intrarenal surgery, cECIRS: conventional endoscopic combined intrarenal surgery, PCNL: percutaneous nephrolithotomy.

The search flow diagram is shown in Figure 1. Seven studies were included in the meta-analysis.

### 3.2. Study Characteristics and Risk of Bias Assessment

Table 1 illustrates the characteristics of the seven included studies [17,18,19,20,21,22,23]. The risk of bias assessment is shown in Appendix D.

### 3.3. Outcomes

#### 3.3.1. Efficacy Outcome

##### Initial Stone Free Rate (Initial SFR)

**Figure 2:**
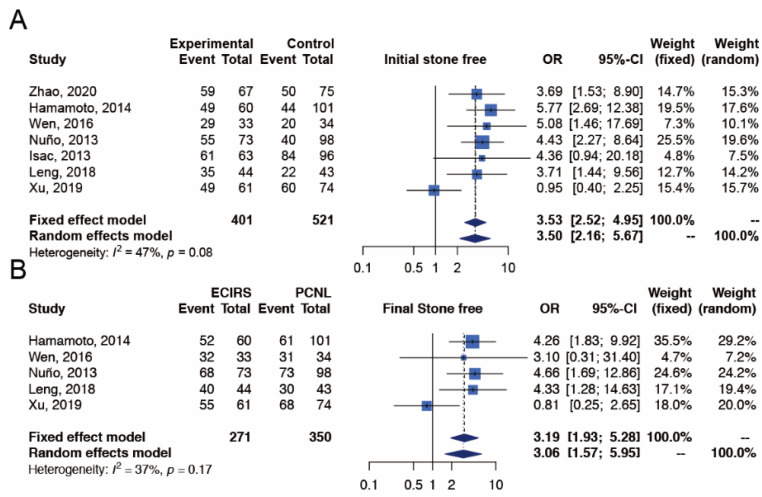
Meta-analysis of efficacy outcomes, including (**A**) initial stone free rate [17,18,19,20,21,22,23] and (**B**) final stone free rate [17,19,20,22,23] between ECIRS and PCNL groups.

The outcome of initial SFR was reported in all seven studies [17,18,19,20,21,22,23], which included 401 and 521 patients in the ECIRS and PCNL groups, respectively (Figure 2). The initial SFR was significantly higher in ECIRS patients than in PCNL patients (random-effects, OR 3.50; 95% CI 2.16–5.67; I^2^  =  47%, Cochran’s Q test *p*-value = 0.08). In TSA, the cumulative number of patients exceeded the required information size of 243 and the Z-curves surpassed the significance boundary in favor of ECIRS, suggesting conclusive results and providing convincing statistical evidence to our findings. The TSA-adjusted confidence interval was OR 3.50 with 95% CI 1.87–6.53 (Appendix J).

##### Final Stone Free Rate (Final SFR)

The outcome of final SFR was reported in five studies [17,19,20,22,23], which included 271 and 350 patients in the ECIRS and PCNL groups, respectively (Figure 2). The final SFR was significantly higher in ECIRS patients than in PCNL patients (random-effects, OR 3.06; 95% CI 1.57–5.59; I^2^  =  37%, Cochran’s Q test *p*-value = 0.17). In TSA, the cumulative number of patients exceeded the required information size of 304 and the Z-curves surpassed the significance boundary in favor of ECIRS, suggesting conclusive results and providing convincing statistical evidence to our findings. The TSA-adjusted confidence interval was OR 3.06 with 95% CI 1.19–7.85 (Appendix J).

#### 3.3.2. Safety Outcome

##### Overall Complications

**Figure 3:**
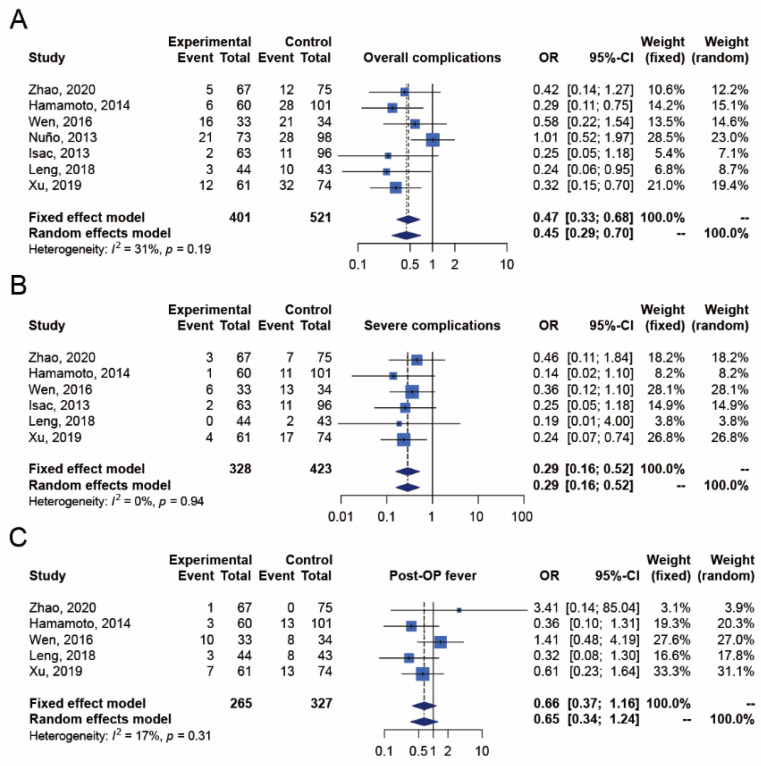
Meta-analysis of safety outcomes, including (**A**) overall complications [17,18,19,20,21,22,23], (**B**) severe complications [17,18,19,21,22,23], and (**C**) postoperative fever [17,18,19,22,23] between ECIRS and PCNL groups.

The overall complication outcome was reported in seven studies [17,18,19,20,21,22,23], which included 401 and 521 patients in the ECIRS and PCNL groups, respectively (Figure 3). Patients with overall complications were significantly fewer in the ECIRS group than in the PCNL group (random-effects, OR 0.45; 95% CI 0.29–0.70; I^2^  =  31%, Cochran’s Q test *p*-value = 0.19). In TSA, the cumulative number of patients exceeded the required information size of 675 and the Z-curves surpassed the significance boundary in favor of ECIRS, suggesting conclusive results and providing convincing statistical evidence to our findings. The TSA-adjusted confidence interval was OR 0.45 with 95% CI 0.28–0.72 (Appendix J).

##### Severe Complications

The outcome of severe complications was reported in six studies [17,18,19,21,22,23], which included 328 and 423 patients in the ECIRS and PCNL groups, respectively (Figure 3). The number of patients with severe complications was significantly fewer in the ECIRS group than in the PCNL group (random-effects, OR 0.29; 95% CI 0.16–0.52; I^2^  = 0%, Cochran’s Q test *p*-value = 0.94). In TSA, the cumulative number of patients exceeded the required information size of 500, and the Z-curves surpassed the significance boundary in favor of ECIRS, suggesting conclusive results and providing convincing statistical evidence to our findings. The TSA-adjusted confidence interval was OR 0.29 with 95% CI 0.15–0.56 (Appendix J).

##### Postoperative Fever

The outcome of postoperative fever was reported in five studies [17,18,19,22,23], which included 265 and 327 patients in the ECIRS and PCNL groups, respectively (Figure 3). The incidence of postoperative fever was not significantly different between ECIRS and PCNL patients (random-effects, OR 0.65; 95% CI 0.34–1.24; I^2^ = 17%, Cochran’s Q test *p*-value = 0.31). In TSA, the cumulative number of patients did not exceed the required information size of 2822, and the Z-curves did not surpass any significance boundary either, suggesting inconclusive results. Further studies are needed to provide convincing statistical evidence. The TSA-adjusted confidence interval was OR 0.65 with 95% CI 0.14–2.97 (Appendix J).

#### 3.3.3. Bleeding Risk

##### Hemoglobin Drop

**Figure 4:**
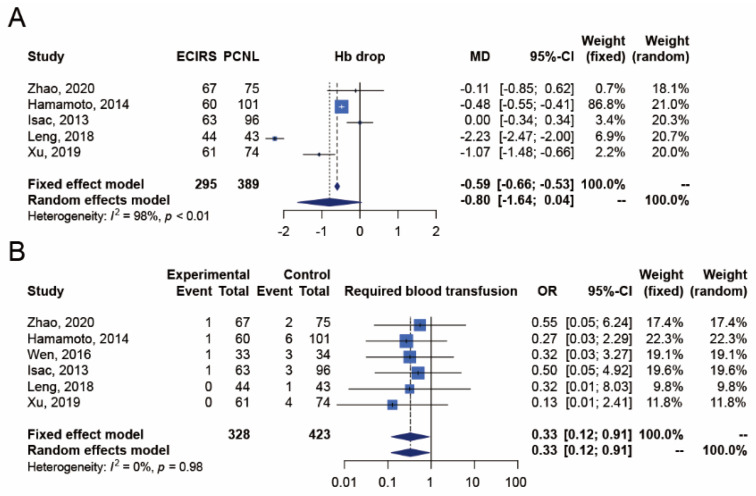
Meta-analysis of bleeding risks, including (**A**) hemoglobin drop [18,19,21,23] and (**B**) required blood transfusion [17,19,21,23] between ECIRS and PCNL groups.

The outcome of hemoglobin drop was reported in five studies [18,19,21,22,23], which included 295 and 389 patients in the ECIRS and PCNL groups, respectively (Figure 4). The incidence of hemoglobin drop was not significantly different between ECIRS and PCNL patients (random-effects, MD −0.80 g/dL; 95% CI −1.64–0.04; I^2^ = 98%, Cochran’s Q test *p* value < 0.01). In TSA, the cumulative number of patients did not exceed the required information size of 1658, and the Z-curves did not surpass any significance boundary either, suggesting inconclusive results. Further studies are needed to provide convincing statistical evidence. The TSA-adjusted confidence interval was MD −0.80 with 95% CI −2.22–0.63 g/dL (Appendix J).

##### Required Blood Transfusion

The outcome of required blood transfusion was reported in six studies [17,18,19,21,22,23], which included 328 and 423 patients in the ECIRS and PCNL groups, respectively (Figure 4). The number of required blood transfusions was lower among ECIRS patients than in PCNL patients (random-effects, OR 0.33; 95% CI 0.12–0.91; I^2^ = 0%, Cochran’s Q test *p*-value 0.98). In TSA, the cumulative number of patients did not exceed the required information size of 799, and the Z-curves only surpassed the traditional significance boundary in favor of ECIRS but not the TSA monitoring boundary, suggesting inconclusive results. Further studies are needed to provide convincing statistical evidence. The TSA-adjusted confidence interval was OR 0.33 with 95% CI 0.10–1.02 (Appendix J).

#### 3.3.4. Efficiency Outcome

##### Operative Time

**Figure 5:**
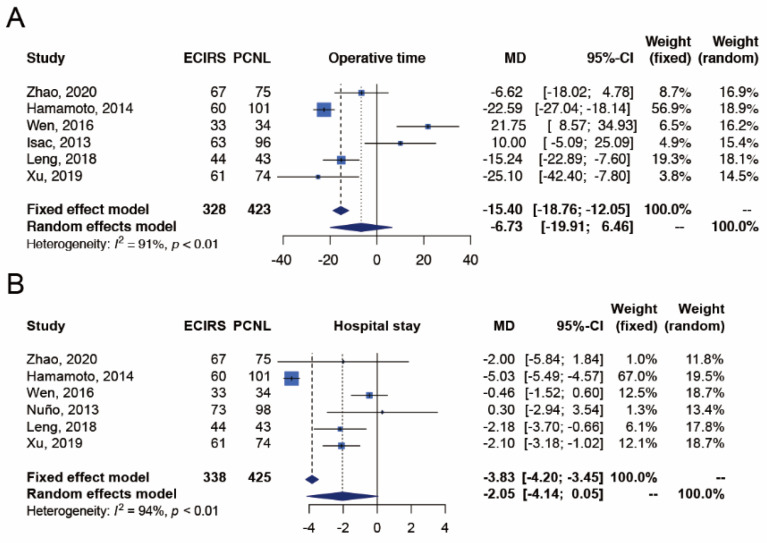
Meta-analysis of efficiency outcomes, including (**A**) operative time [17,18,19,21,22,23] and (**B**) hospital stay [17,18,19,20,22,23] between ECIRS and PCNL groups.

The outcome of operative time was reported in six studies [17,18,19,21,22,23], which included 328 and 423 patients in the ECIRS and PCNL groups, respectively (Figure 5). Operative time was not significantly different between ECIRS and PCNL patients (random-effects, MD −6.73 min; 95% CI −19.91–6.46; I^2^  = 91%, Cochran’s Q test *p*-value < 0.01). In TSA, the cumulative number of patients did not exceed the required information size of 5901, and the Z-curves did not surpass any significance boundary either, suggesting inconclusive results. Further studies are needed to provide convincing statistical evidence. The TSA-adjusted confidence interval was MD −6.73 with 95% CI −60.55–47.10 min (Appendix J).

##### Hospital Stay

The outcome of hospital stay was reported in six studies [17,18,19,20,22,23], which included 338 and 425 patients in the ECIRS and PCNL groups, respectively (Figure 5). The length of hospital stay was not significantly different between ECIRS and PCNL patients (random-effects, MD −2.05 days; 95% CI −4.14–0.05; I^2^  = 94%, Cochran’s Q test *p*-value < 0.01). In TSA, the cumulative number of patients did not exceed the required information size of 1646 and the Z-curves did not surpass any significance boundary either, suggesting inconclusive results. Further studies are needed to provide convincing statistical evidence. The TSA-adjusted confidence interval was MD −2.05 with 95% CI −5.37–1.28 days (Appendix J).

### 3.4. Subgroup Analysis in Different Procedure Types and Study Types

In subgroup analysis, patients receiving mECIRS versus mPCNL (Appendix F) had higher initial SFR, higher final SFR, fewer overall complications, fewer severe complications, shorter hospital stay and lower incidence of postoperative fever, but no difference in operative time, incidence of hemoglobin drop and patients requiring blood transfusion. Besides, in the subgroup analysis of different study types (Appendix G), the results from retrospective cohort studies did not alter the trend of meta-analysis results in all the outcomes.

### 3.5. Meta-Regression

In the meta-regression, there was no difference in the interaction of the publication date and radius access length with all the outcomes, which indicated that the heterogeneities of the publication date (Appendix K) and Amplatz sheath size (Appendix L) did not influence the results of meta-analysis.

### 3.6. Sensitivity Analysis

In the sensitivity analysis, the pooled estimates within the 95% CI were maintained after excluding the highest risk-of-bias cohort studies (i.e., Newcastle–Ottawa quality assessment score ≤ 7) across all the results for these outcomes (Appendix H).

Furthermore, we performed a stepwise sensitivity analysis to exclude the high risk-of-bias cohort studies (i.e., Newcastle–Ottawa quality assessment score ≤ 8). The pooled estimates within the 95% CI were maintained across all the results for these outcomes, except for required blood transfusion (Appendix I).

## 4. Discussion

To our knowledge, this is the first systematic review and meta-analysis to investigate and compare the efficacy, safety, and efficiency of ECIRS and PCNL on patients with complex renal stones. We found that ECIRS improved both initial and final SFR, while lowering both overall and severe complications as well as the need for blood transfusion. No significant differences were found for the other complications (i.e., postoperative fever and hemorrhage) as well as for operative time and hospital stay.

Performing additional RIRS at the same time of PCNL contribute to the improvement of SFR by serving diagnostic and therapeutic functions, including supervision of renal access and the urinary tract below the kidney, avoidance of multiple percutaneous access tracks by endoscopic exploration of calices that were unreachable by nephroscopy, irrigation during lithotripsy, and passing the stone fragments through the Amplatz sheath [3].

To depict the cooperative relationship between RIRS and PCNL more vividly, the following example can be made. Two cases (2.7%) of patients undergoing PCNL monotherapy presented with steinstrasse, multiple stone fragments accumulating along the ureter after the surgery, in the study by F Zhao et al., while none was found in the ECIRS group [18]. In ECIRS, the fragments in the ureter could be pushed upward and extracted through the Amplatz sheath when coordinating the two pieces of equipment. Performed together, RIRS and PCNL have a synergistic effect and overcome their individual limitations.

The most concerning complications of PCNL are hemorrhage, infection, and thoracic complications (i.e., pneumothorax, hemothorax, etc.) [24]. RIRS concerns people the most with ureteral stent discomfort, ureteral wall injury, and stone migration [25]. Some may argue that ECIRS can possibly add up the risks of both PCNL and RIRS [4]; in fact, in our meta-analysis, ECIRS had significantly fewer overall and severe complications than PCNL. ECIRS patients also required less blood transfusions. As more excessive bleeding conditions necessitate more blood transfusions [26], our meta-analysis suggested that ECIRS causes fewer massive bleeding events. Compared with PCNL, the mean difference of hemoglobin drop in ECIRS group is −0.8 (−1.64; 0.04), which suggests there may be less blood loss. Although there are no significant differences, Zhao et al., Hamamoto et al., Leng et al., and Xu et al. all support such trend.

Subgroup analyses were performed to compare between the conventional group and the minimally invasive group. In 1976, Fernström and Johansson first invented cPCNL, also termed standard PCNL, which has a tract size ≥22 Fr [24]. On the other hand, in 1998, Jackman introduced the miniaturization of the instrument set (now termed mPCNL) for the treatment of nephrolithiasis in children then [27]. In our subgroup analysis (mECIRS vs mPCNL; cECIRS vs cPCNL), the SFR and complication rate were consistent with the primary outcome (ECIRS vs PCNL). In the minimally invasive subgroup (mECIRS vs mPCNL), mECIRS had significantly shorter hospital stays, according to de la Rosette, which was associated with lower Clavien-Dindo scores, implying fewer severe complications [28]. Moreover, mECIRS requiring fewer auxiliary procedures may also shorten hospital stay [18].

However, ECIRS is still not prevalent in clinical practice due to several concerns. First, requiring two endovision systems and cooperation between two surgeons can be an issue in limited-resource settings. Second, the problem of cost was mentioned in the study by Jung HD et al., wherein cases of unilateral renal stones are not allowed to require the cost of PCNL and RIRS at the same time, which may be a burden to the hospital in Korea [29]. In Taiwan, ECIRS costs an additional surgical fee and self-paid medical devices that are not covered by the national health insurance; this may influence the patients’ willingness to undergo the surgery. Third, with the combination of two procedures, operative time is sometimes considered longer in ECIRS. In fact, our meta-analysis revealed no significant difference in operative time between ECIRS and PCNL [17,20]. The concerns mentioned above should be reevaluated as we came to realize: the elimination of potential complications decreases the expense of rescue measures, such as blood transfusion or intravenous antibiotics, and the superior SFR eliminates the need for auxiliary procedures and their associated costs [30]. The advantages of ECIRS may outweigh its disadvantages to some extent.

In our meta-analysis, we have not only carefully screened and included the studies that met the aforementioned criteria, but also performed subgroup analyses to clarify the differences among minimally invasive and conventional groups separately. However, our study has some limitations. First, the number of patients included (*n* = 919) was relatively small, which may result from the fact that ECIRS is still not clinically prevalent. Second, six out of the seven included studies were not RCTs, which can possibly cause intrinsic bias. There was also no consensus on the patient’s position (prone, supine, or GMSV position) [3]. Heterogeneity may exist among the included studies. There was a paucity of RCTs that could have elucidated the comparative outcomes of ECIRS and other methods to remove complex renal stones. More detailed secondary outcomes can be obtained in future studies to cover our limitations.

## 5. Conclusions

In conclusion, our meta-analysis of the current evidence suggests that ECIRS is more effective and safer than PCNL. When treating complex renal stones, ECIRS has better initial/final SFR, fewer overall/severe complications, and requires fewer blood transfusions than PCNL. Both minimally invasive and conventional subgroups supported ECIRS in the SFR and complication outcomes. In the minimally invasive subgroup, ECIRS was favored due to shorter hospital stays and less postoperative fever. No significant differences were found in other outcomes, which require more high-quality studies to determine.

## Figures and Tables

**Table 1:** Characteristics of included studies.

Author, Year	Country	Study Period	Study Design	No. of Patients	Age (Mean)	Male (%)	BMI (kg/m^2^)	Stone Burden Characteristics	No. of Staghorn Stone (%)	No. of Complete Staghorn stone (%)	Intervention	Comparator	Percutaneous Access Size	ECIRS Position	PCNL Position
Zhao, 2020 [17]	China	Jan 2018– Oct 2019	RCS	140	53.13	64.2	25.61	Area 700 mm^2^	16.4	8.4	mECIRS	mPCNL	16–18F	GMSV	prone
Hamamoto, 2014 [18]	Japan	Feb 2004– Jan 2013	RCS	161	53.17	75.8	24.62	Max 36.7 mm	35.4	17.4	mECIRS	mPCNL, cPCNL	(mini) 18F, (con) 30F	prone split-leg	prone
Wen, 2016 [19]	China	May 2012– Oct 2014	RCT	67	44.49	58.2	21.9	Area 667 mm^2^	100	NS	mECIRS	mPCNL	20F	GMSV	prone
Nuño, 2013 [20]	Spain	Jan 2005– Dec 2011	RCS	171	51.4	42.1	NS	Area 694.1 mm^2^	43.2	24.6	cECIRS	cPCNL	24–30F	GMSV	supine
Isac, 2013 [21]	USA	Aug 2010– Jan 2012	RCS	158	57.6	45.5	30.78	Cumulative 30.6 mm	NS	NS	cECIRS	cPCNL	30F	prone split-leg	prone
Leng, 2018 [22]	Japan	Feb 2004– Jan 2013	RCS	87	45.98	59.8	NS	Mean 52.2 mm	100	33.3	mECIRS	mPCNL	16–18F	oblique supine lithotomy	oblique supine lithotomy
Xu, 2019 [23]	China	NS	RCS	135	50.03	48.2	23.05	Mean 58.14 mm	100	65.19	mECIRS	mPCNL	16–22F	NS	NS

RCS: retrospective cohort studies; RCT: randomized control trial; mECIRS: minimally-invasive endoscopic combined intrarenal surgery; cECIRS: conventional endoscopic combined intrarenal surgery; mPCNL: minimally-invasive percutaneous nephrolithotomy; cPCNL: conventional percutaneous nephrolithotomy; F: French; GMSV: Galdakao-modified supine Valdivia; NS: not specified.

**Figure A1:**
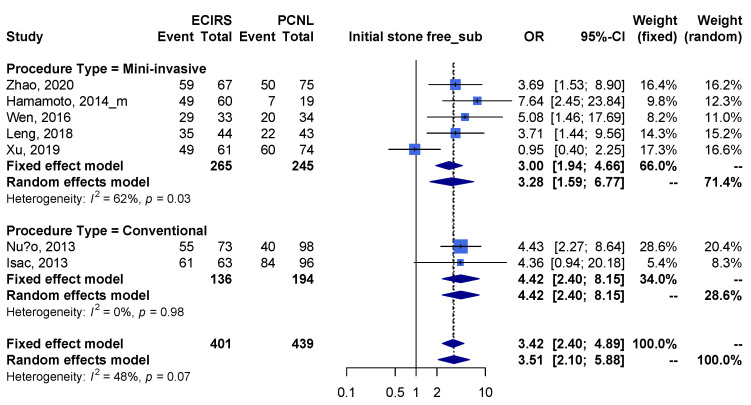
Subgroup analysis of outcome regarding initial stone free rate. (Mini: [17,18,19,22,23]; Conventional: [20,21]).

**Figure A2:**
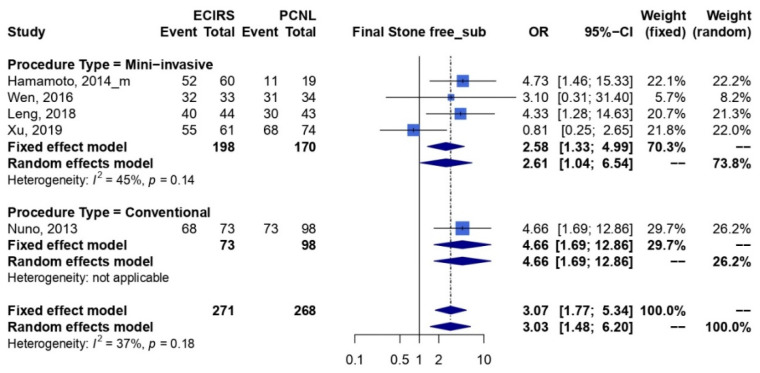
Subgroup analysis of outcome regarding final stone free rate. (Mini: [17,19,22,23]; Conventional: [20]).

**Figure A3:**
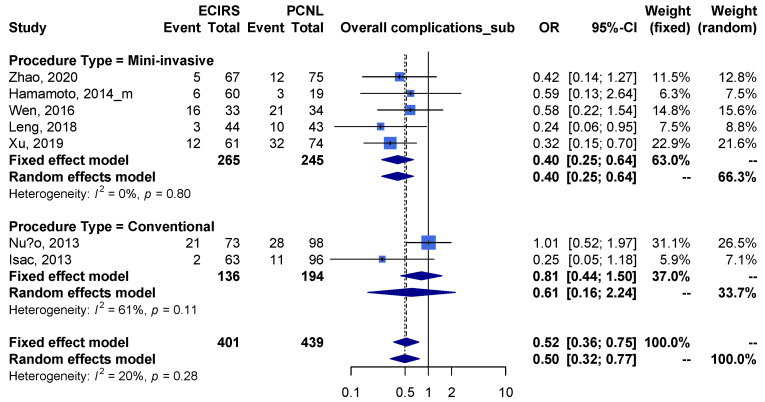
Subgroup analysis of outcome regarding overall complications. (Mini: [17,18,19,22,23]; Conventional: [20,21]).

**Figure A4:**
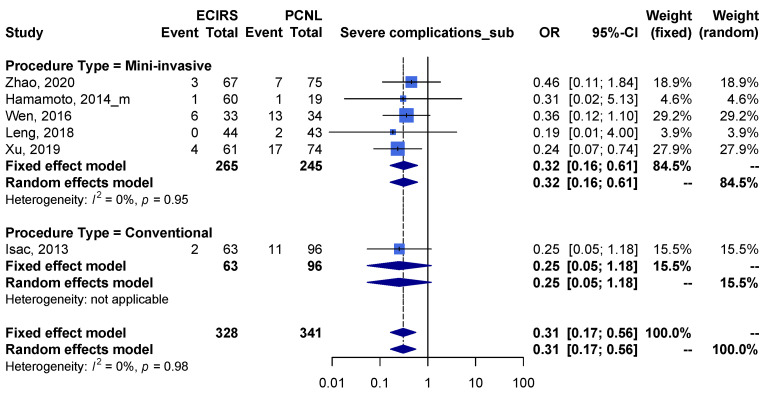
Subgroup analysis of outcome regarding severe complications. (Mini: [17,18,19,22,23]; Conventional: [21]).

**Figure A5:**
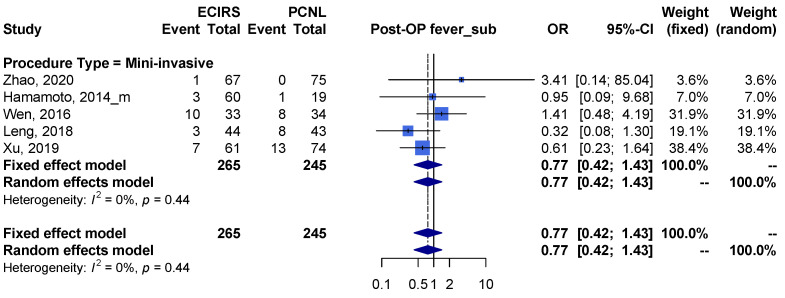
Subgroup analysis of outcome regarding post-operative fever. (Mini: [17,18,19,22,23]).

**Figure A6:**
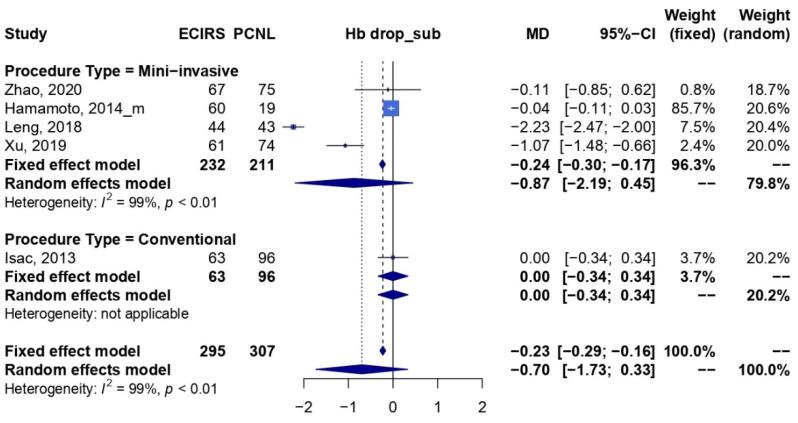
Subgroup analysis of outcome regarding hemoglobin drop. (Mini: [18,19,22,23]; Conventional: [21]).

**Figure A7:**
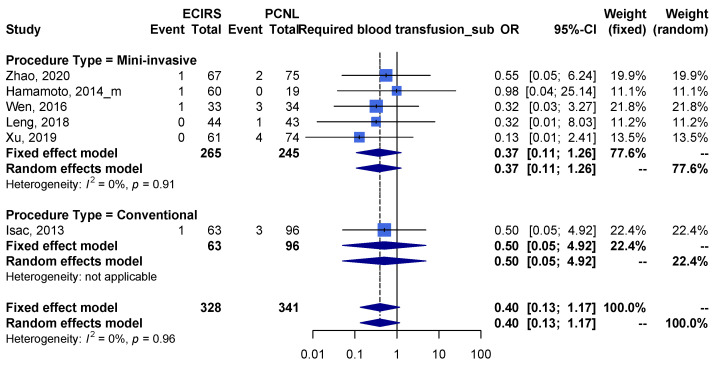
Subgroup analysis of outcome regarding required blood transfusion. (Mini: [17,18,19,22,23]; Conventional: [21]).

**Figure A8:**
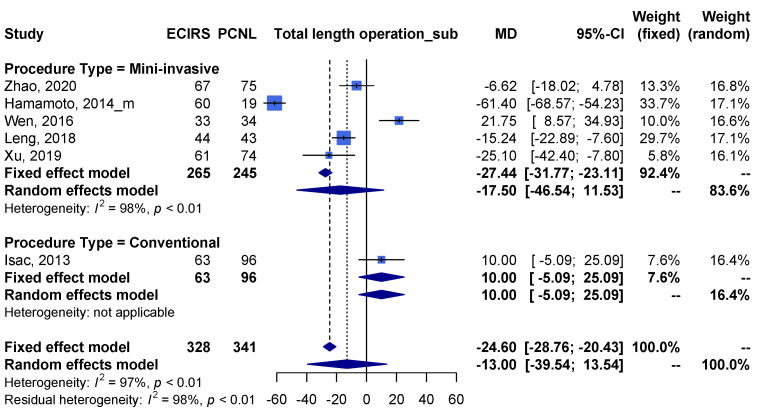
Subgroup analysis of outcome regarding operative time (Mini: [17,18,19,22,23]; Conventional: [21]).

**Figure A9:**
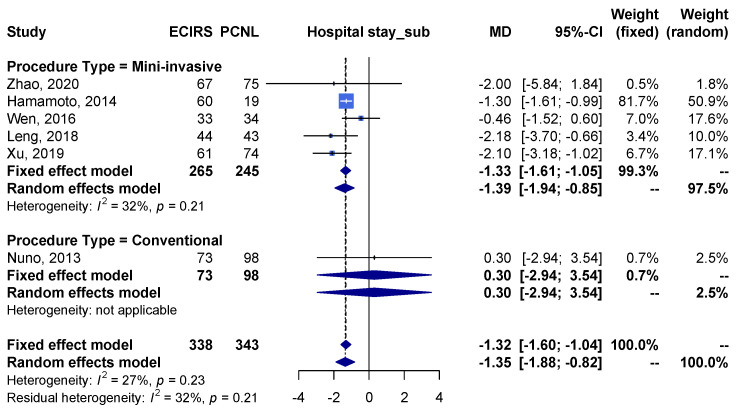
Subgroup analysis of outcome regarding hospital stay. (Mini: [17,18,19,22,23]; Conventional: [20]).

**Figure A10:**
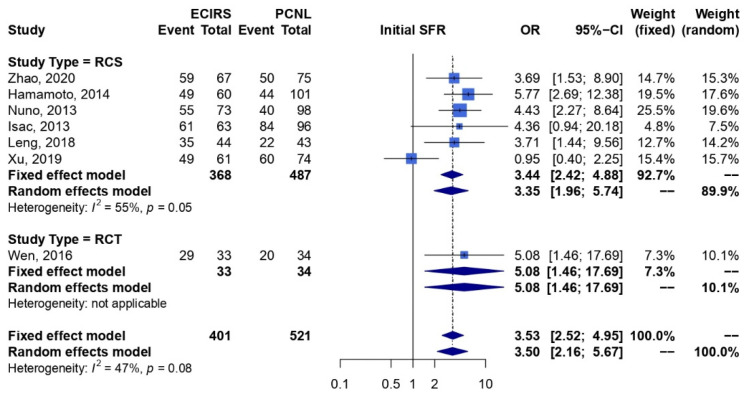
Subgroup analysis of outcome regarding initial stone free rate. (RCS: [18,19,20,21,22,23]; RCT: [17]).

**Figure A11:**
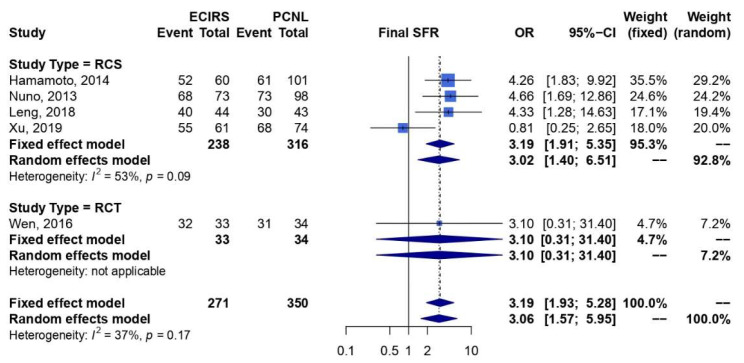
Subgroup analysis of outcome regarding final stone free rate. (RCS: [18,19,20,21,22,23]; RCT: [17]).

**Figure A12:**
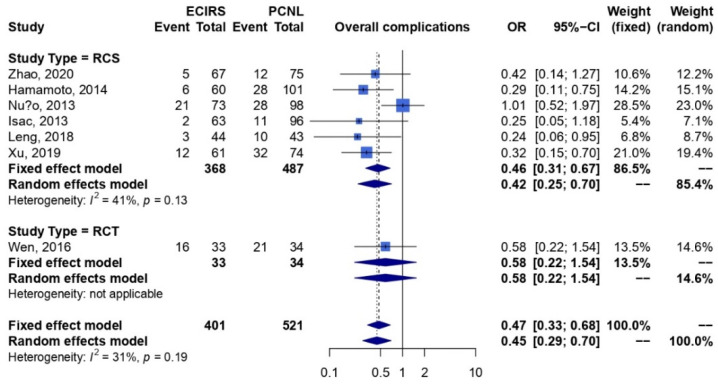
Subgroup analysis of outcome regarding overall complications. (RCS: [18,19,20,21,22,23]; RCT: [17]).

**Figure A13:**
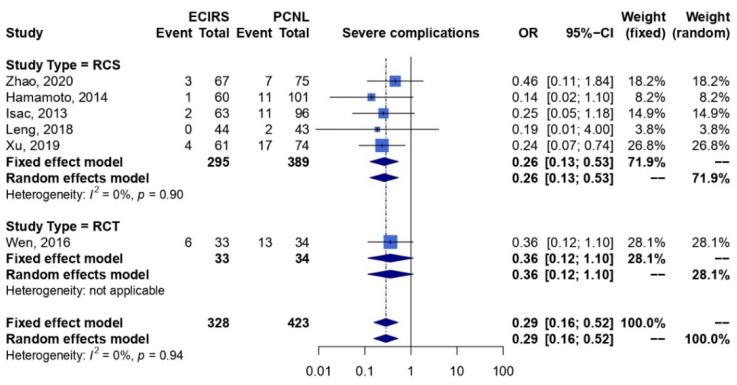
Subgroup analysis of outcome regarding severe complications. (RCS: [18,19,20,21,22,23]; RCT: [17]).

**Figure A14:**
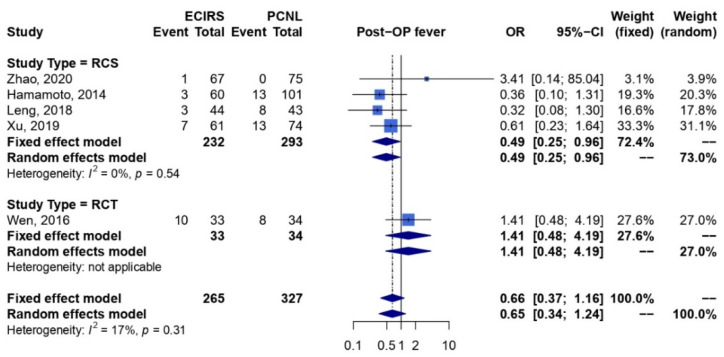
Subgroup analysis of outcome regarding post-operative fever. (RCS: [18,19,20,21,22,23]; RCT: [17]).

**Figure A15:**
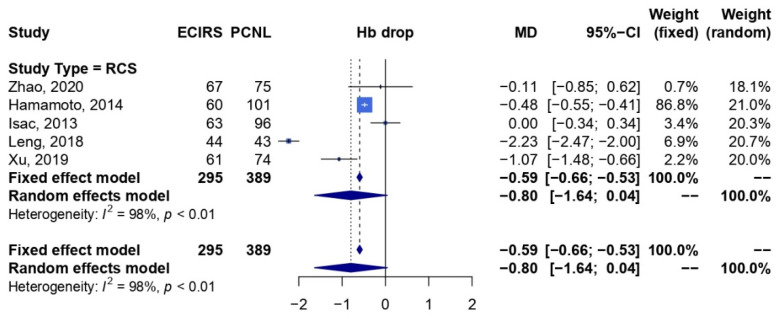
Subgroup analysis of outcome regarding hemoglobin drop. (RCS: [18,19,21,22,23]).

**Figure A16:**
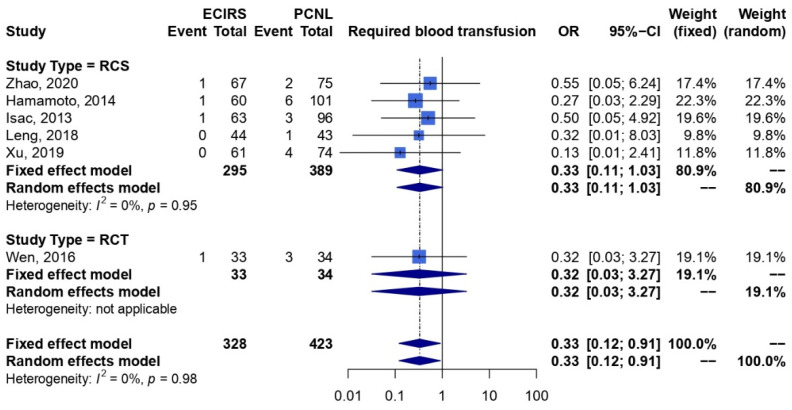
Subgroup analysis of outcome regarding required blood transfusion. (RCS: [18,19,21,22,23]; RCT: [17]).

**Figure A17:**
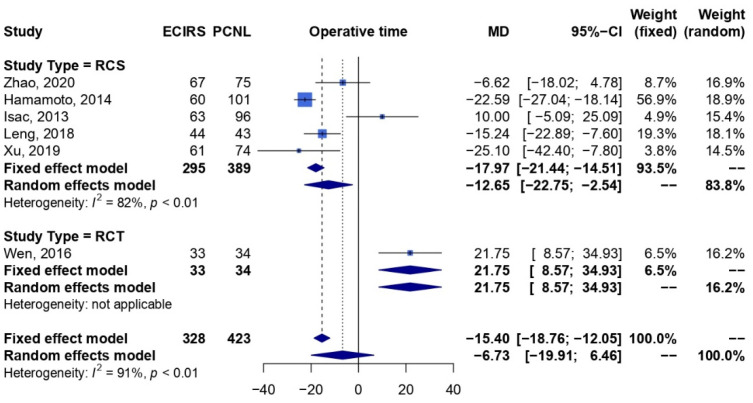
Subgroup analysis of outcome regarding operative time. (RCS: [18,19,21,22,23]; RCT: [17]).

**Figure A18:**
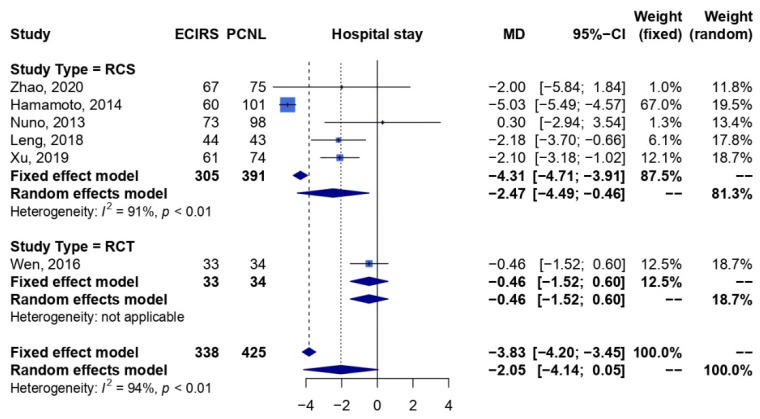
Subgroup analysis of outcome regarding hospital stay. (RCS: [18,19,21,22,23]; RCT: [17]).

**Figure A19:**
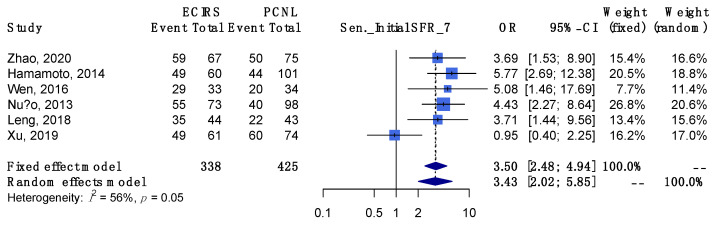
Sensitivity analysis of outcome regarding initial stone free rate. ([17,18,19,20,22,23]).

**Figure A20:**
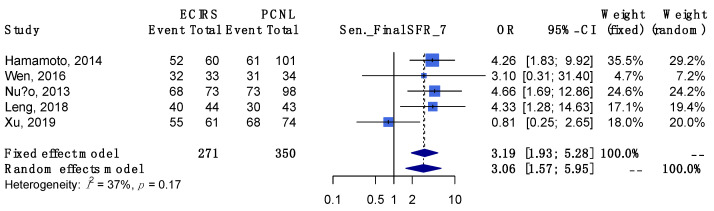
Sensitivity analysis of outcome regarding final stone free rate. ([17,19,20,22,23]).

**Figure A21:**
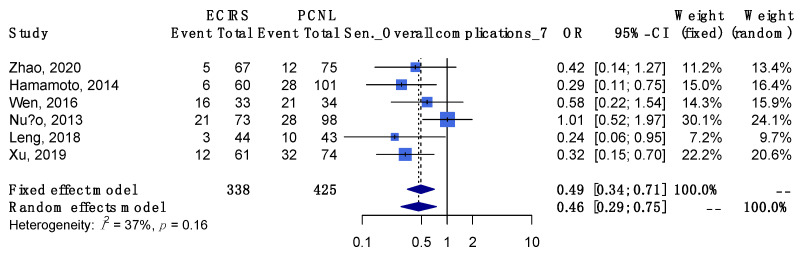
Sensitivity analysis of outcome regarding overall complications. ([17,18,19,20,22,23]).

**Figure A22:**
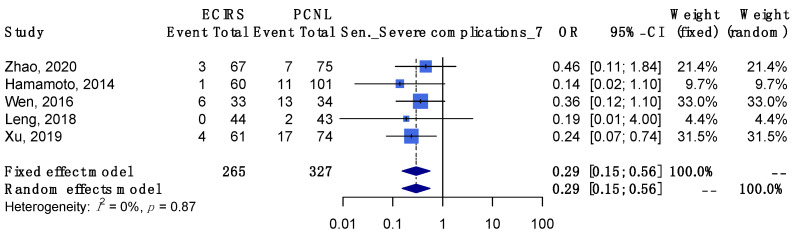
Sensitivity analysis of outcome regarding severe complications. ([17,18,19,22,23]).

**Figure A23:**
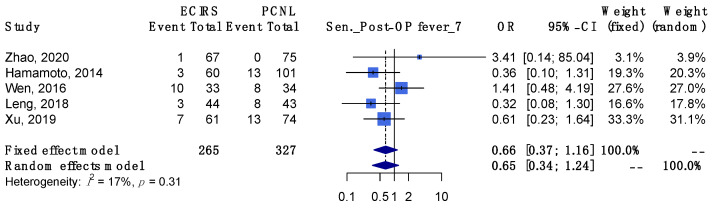
Sensitivity analysis of outcome regarding post-operative fever. ([17,18,19,22,23]).

**Figure A24:**
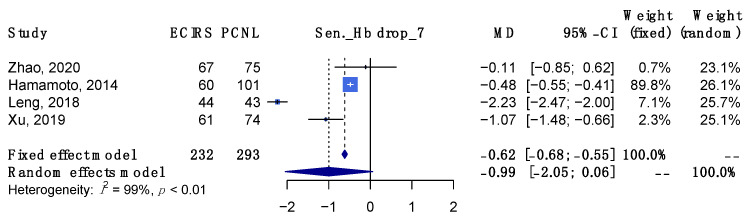
Sensitivity analysis of outcome regarding hemoglobin drop. ([18,19,22,23]).

**Figure A25:**
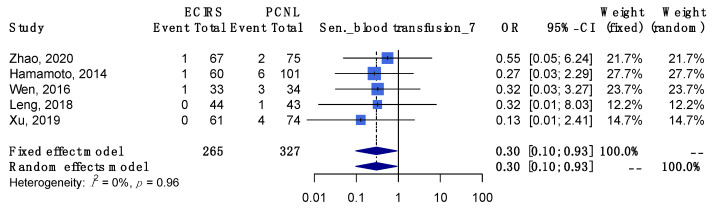
Sensitivity analysis of outcome regarding required blood transfusion. ([17,18,19,22,23]).

**Figure A26:**
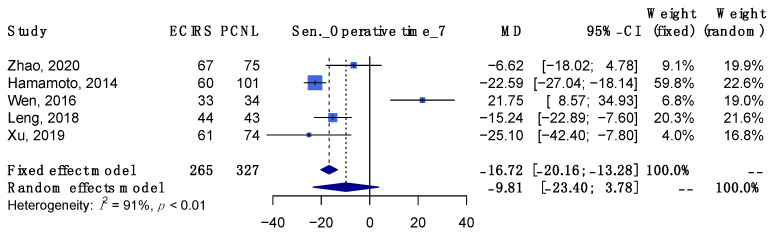
Sensitivity analysis of outcome regarding operative time. ([17,18,19,22,23]).

**Figure A27:**
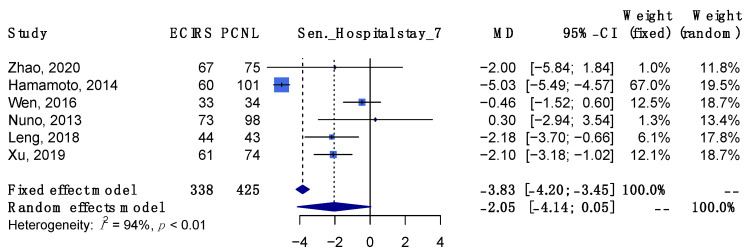
Sensitivity analysis of outcome regarding hospital stay. ([17,18,19,20,22,23]).

**Figure A28:**
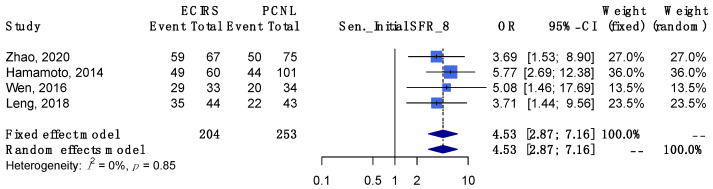
Sensitivity analysis of outcome regarding initial stone free rate. ([17,18,19,22]).

**Figure A29:**
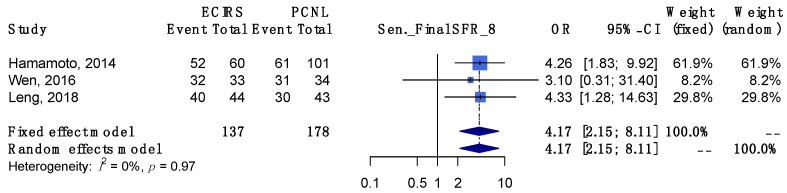
Sensitivity analysis of outcome regarding final stone free rate. ([17,19,22]).

**Figure A30:**
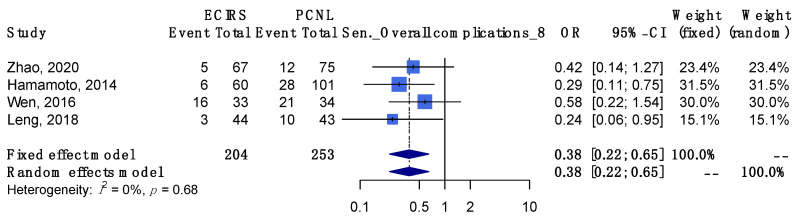
Sensitivity analysis of outcome regarding overall complications. ([17,18,19,22]).

**Figure A31:**
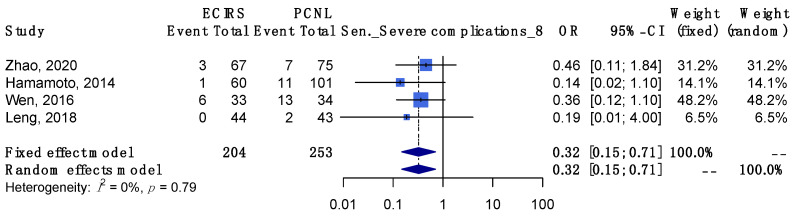
Sensitivity analysis of outcome regarding severe complications. ([17,18,19,22]).

**Figure A32:**
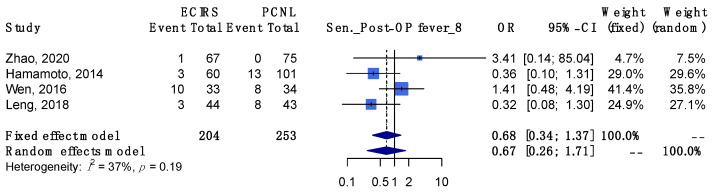
Sensitivity analysis of outcome regarding post-operative fever. ([17,18,19,22]).

**Figure A33:**
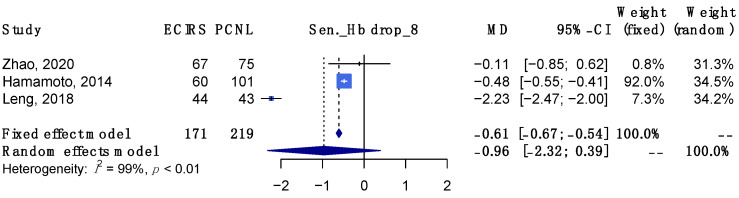
Sensitivity analysis of outcome regarding hemoglobin drop. ([18,19,22]).

**Figure A34:**
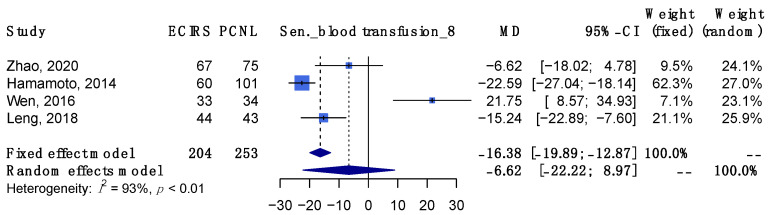
Sensitivity analysis of outcome regarding required blood transfusion. ([17,18,19,22]).

**Figure A35:**
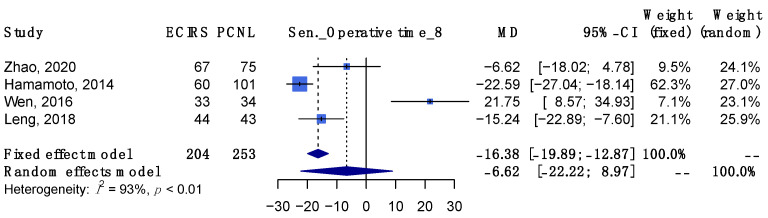
Sensitivity analysis of outcome regarding operative time. ([17,18,19,22]).

**Figure A36:**
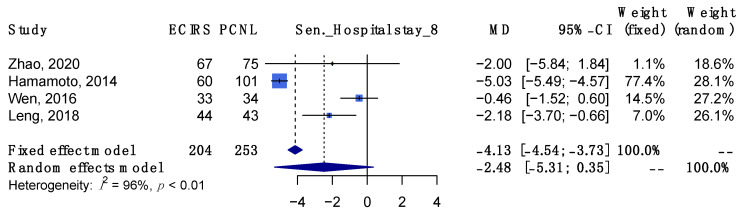
Sensitivity analysis of outcome regarding hospital stay. ([17,18,19,22]).

**Figure A37:**
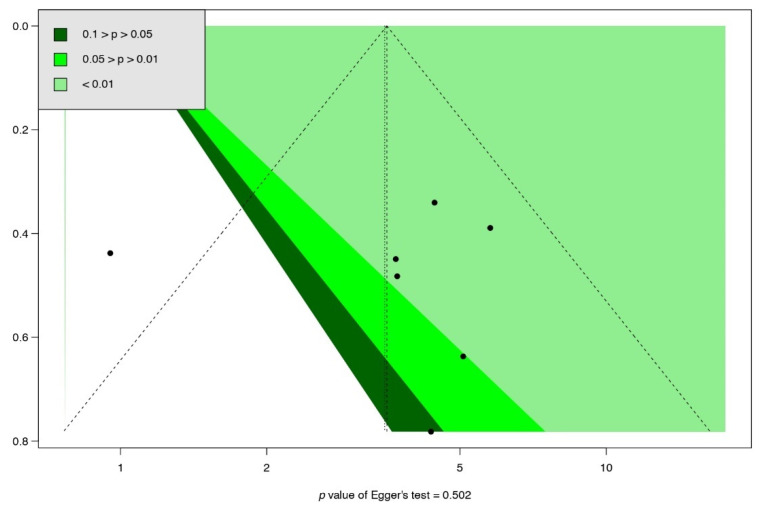
Comparison-adjusted funnel plot in outcome for initial stone free rate.

**Figure A38:**
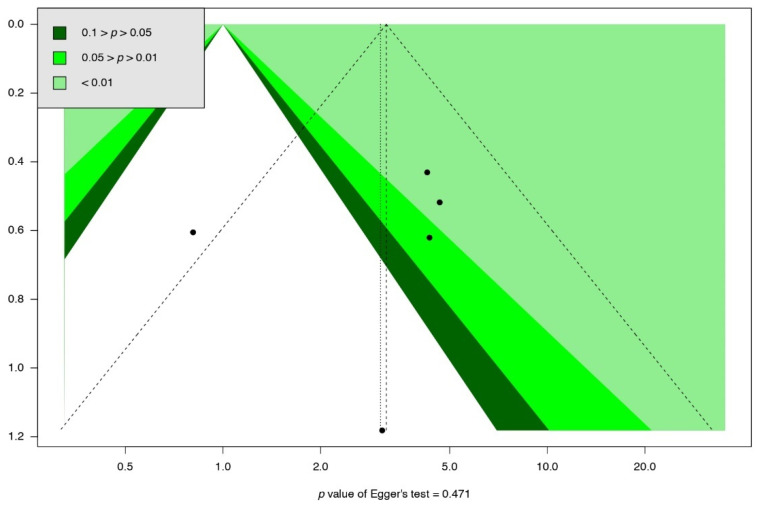
Comparison-adjusted funnel plot in outcome for final stone free rate.

**Figure A39:**
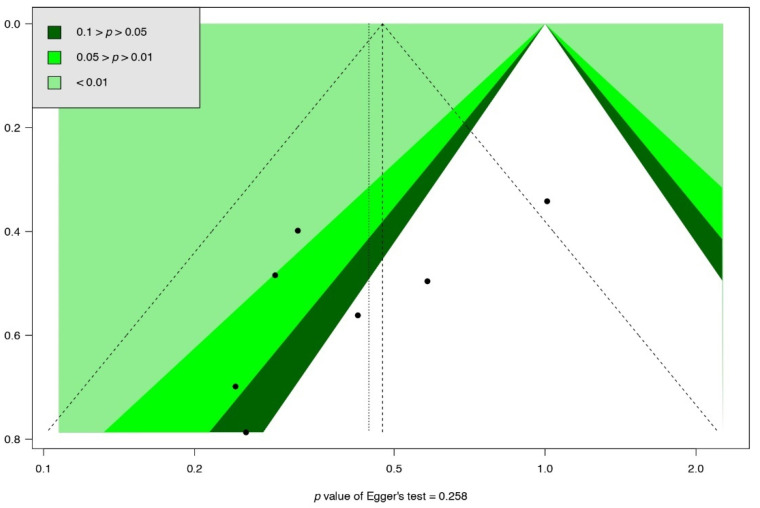
Comparison-adjusted funnel plot in outcome for overall complications.

**Figure A40:**
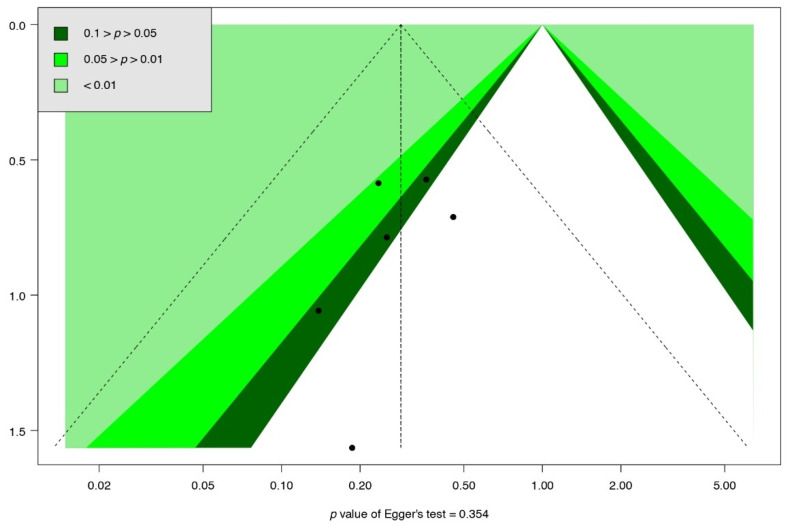
Comparison-adjusted funnel plot in outcome for severe complications.

**Figure A41:**
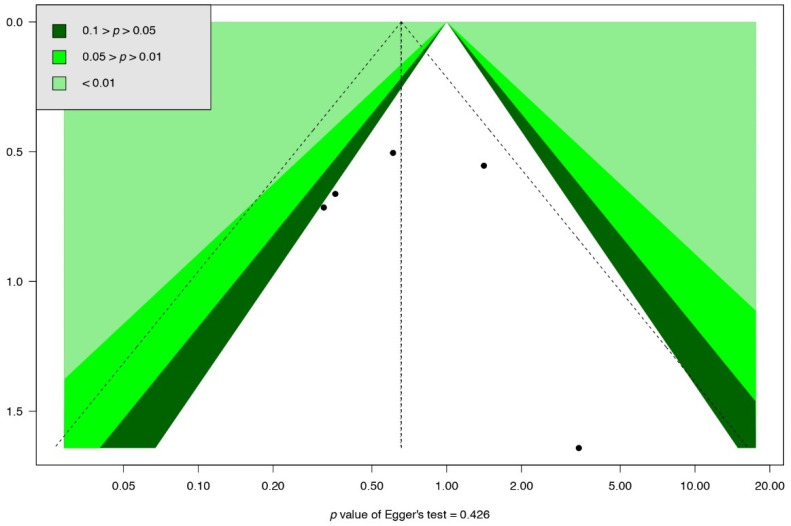
Comparison-adjusted funnel plot in outcome for post-operative fever.

**Figure A42:**
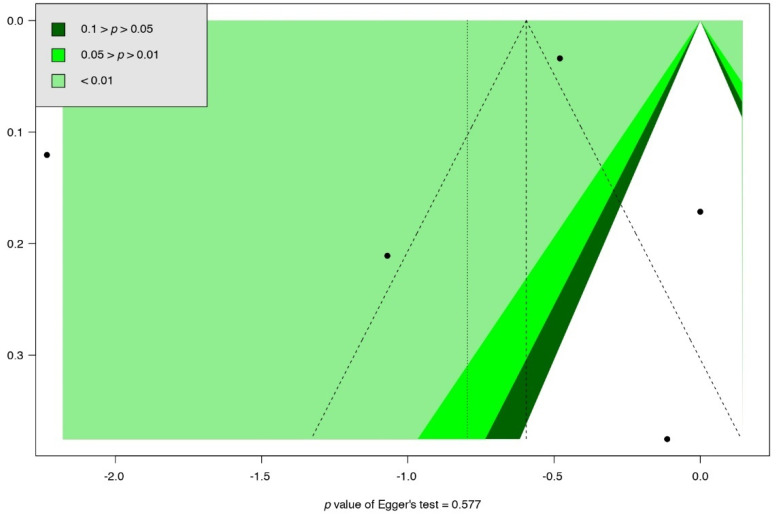
Comparison-adjusted funnel plot in outcome for hemoglobin drop.

**Figure A43:**
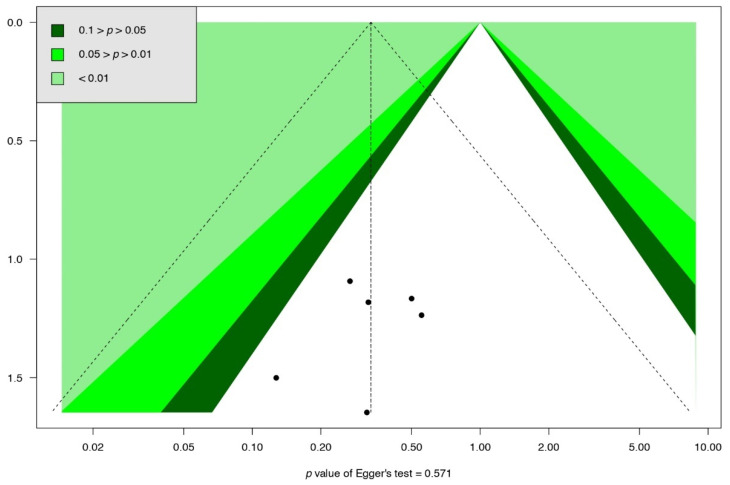
Comparison-adjusted funnel plot in outcome for required blood transfusion.

**Figure A44:**
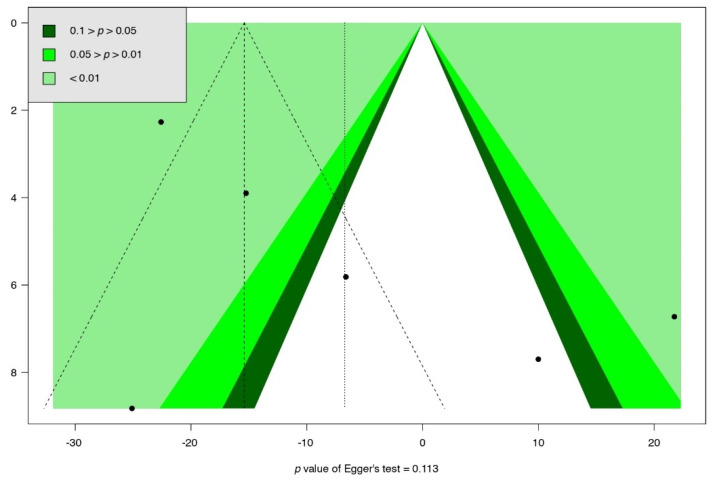
Comparison-adjusted funnel plot in outcome for operative time.

**Figure A45:**
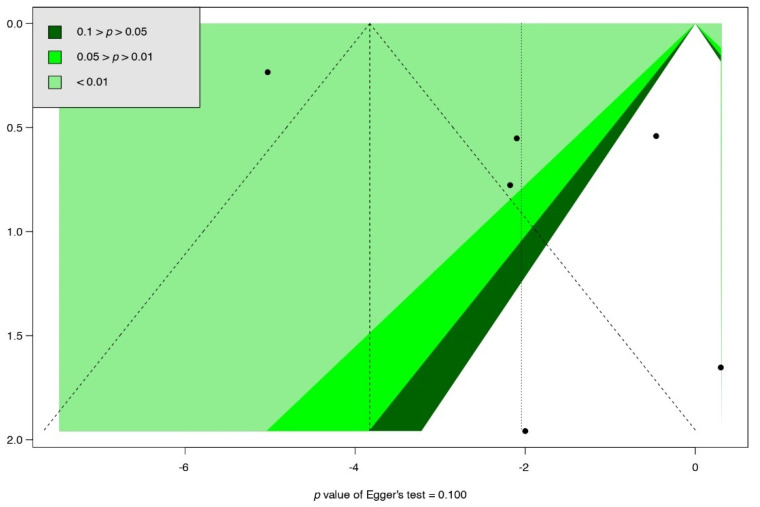
Comparison-adjusted funnel plot in outcome for hospital stay.

**Figure A46:**
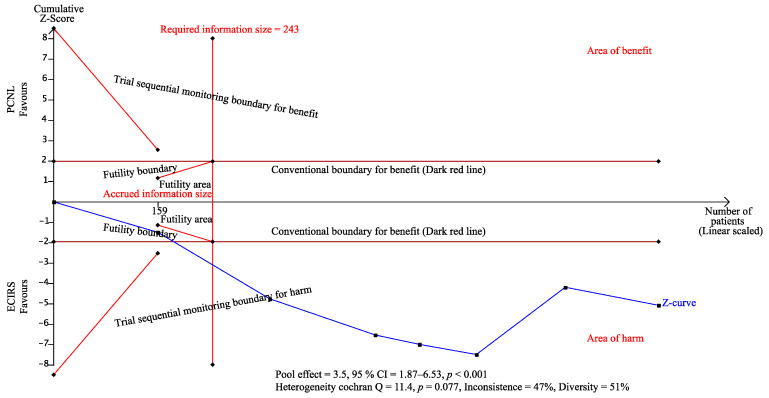
Trial sequential analysis of outcome regarding initial stone free rate.

**Figure A47:**
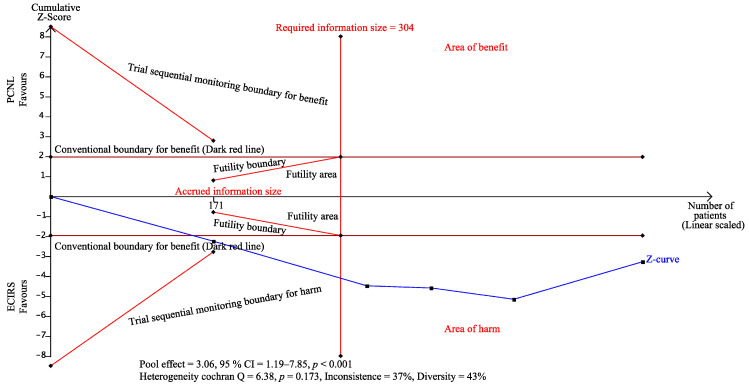
Trial sequential analysis of outcome regarding final stone free rate.

**Figure A48:**
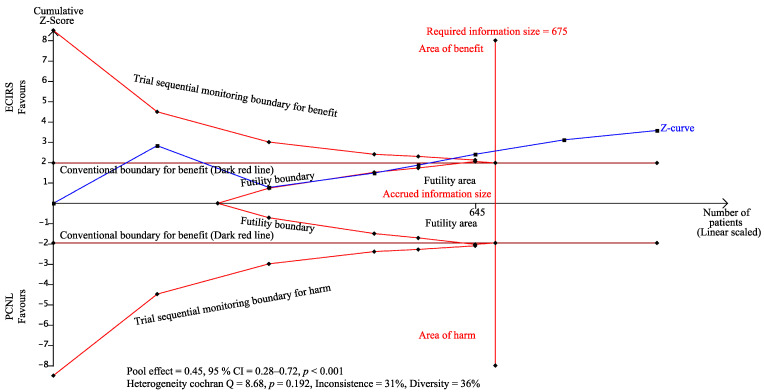
Trial sequential analysis of outcome regarding overall complications.

**Figure A49:**
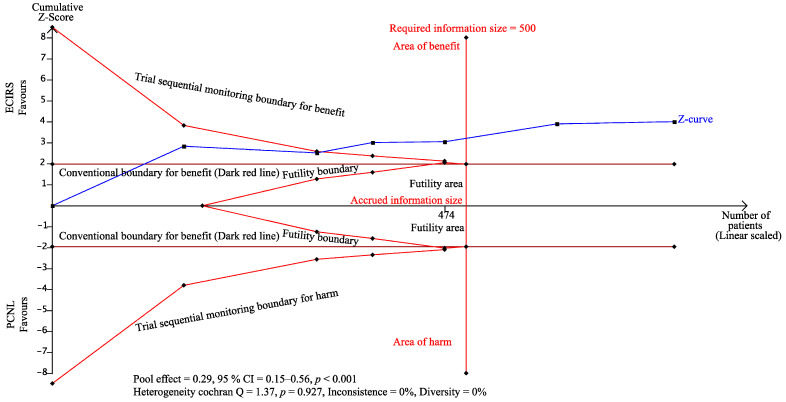
Trial sequential analysis of outcome regarding severe complications.

**Figure A50:**
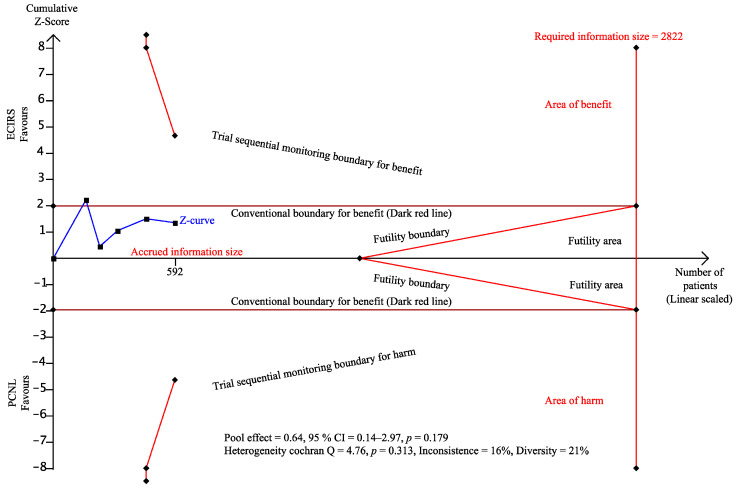
Trial sequential analysis of outcome regarding post-operative fever.

**Figure A51:**
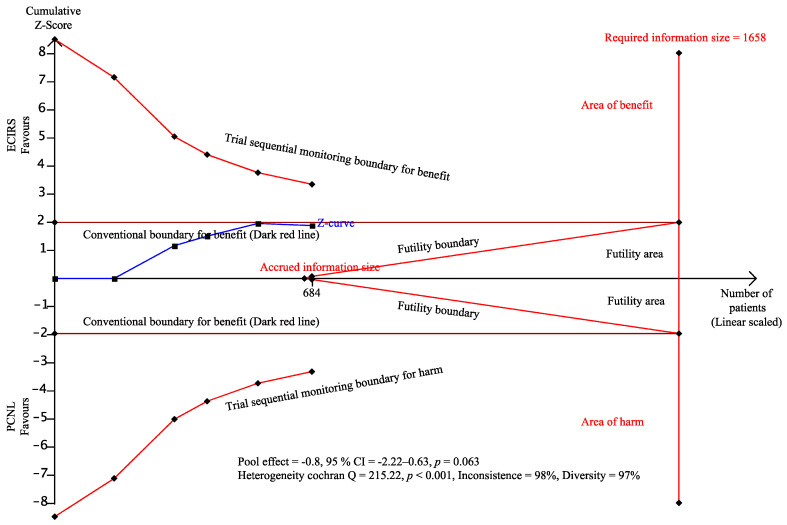
Comparison-adjusted funnel plot in outcome for hemoglobin drop.

**Figure A52:**
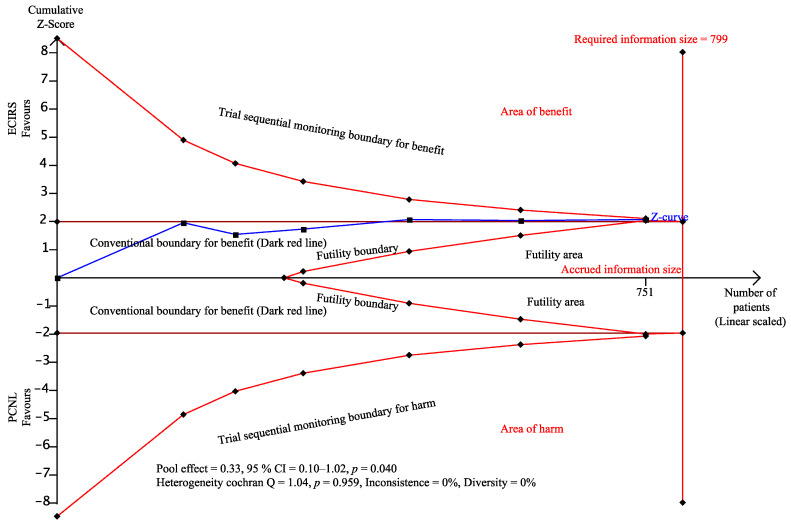
Trial sequential analysis of outcome regarding required blood transfusion.

**Figure A53:**
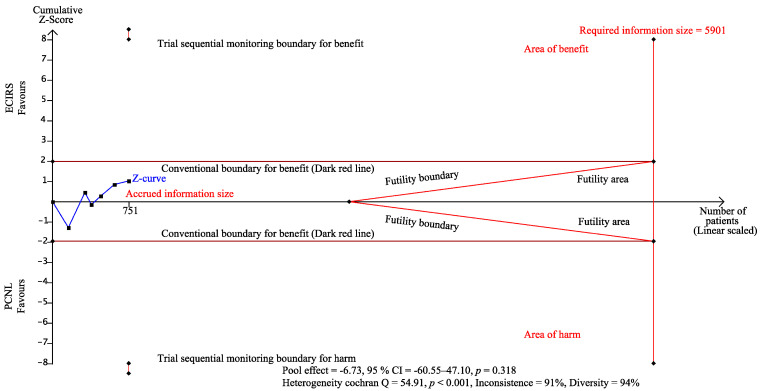
Comparison-adjusted funnel plot in outcome for operative time.

**Figure A54:**
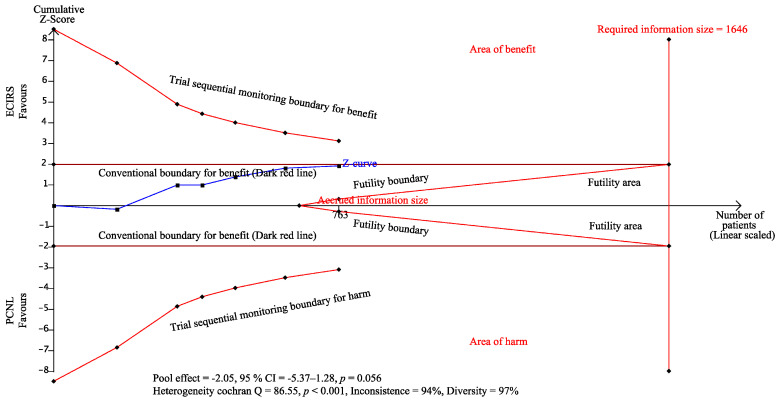
Comparison-adjusted funnel plot in outcome for hospital stay.

## Data Availability

Details of the checklists, literature search strategy, risk of bias assessment, subgroup analyses, sensitivity analyses, and trial sequential analysis are reported in the appendix.

## Appendix A. PRISMA Checklist

**Table A1:** PRISMA—main checklist.

Topic	No.	Item	Location where Item is Reported
**TITLE**			
Title	1	Identify the report as a systematic review.	page 1
**ABSTRACT**			
Abstract	2	See the PRISMA 2020 for Abstracts checklist (Table A2)	
**INTRODUCTION**			
Rationale	3	Describe the rationale for the review in the context of existing knowledge.	page 1–2
Objectives	4	Provide an explicit statement of the objective(s) or question(s) the review addresses.	page 2
**METHODS**			
Eligibility criteria	5	Specify the inclusion and exclusion criteria for the review and how studies were grouped for the syntheses.	page 2
Information sources	6	Specify all databases, registers, websites, organisations, reference lists, and other sources searched or consulted to identify studies. Specify the date when each source was last searched or consulted.	page 2
Search strategy	7	Present the full search strategies for all databases, registers, and websites, including any filters and limits used.	Appendix C
Selection process	8	Specify the methods used to decide whether a study met the inclusion criteria of the review, including how many reviewers screened each record and each report retrieved, whether they worked independently, and, if applicable, details of automation tools used in the process.	page 2
Data collection process	9	Specify the methods used to collect data from reports, including how many reviewers collected data from each report, whether they worked independently, any processes for obtaining or confirming data from study investigators, and, if applicable, details of automation tools used in the process.	page 2
Data items	10a	List and define all outcomes for which data were sought. Specify whether all results that were compatible with each outcome domain in each study were sought (e.g., for all measures, time points, analyses), and if not, the methods used to decide which results to collect.	page 2–3
	10b	List and define all other variables for which data were sought (e.g., participant and intervention characteristics, funding sources). Describe any assumptions made about any missing or unclear information.	page 3
Study risk of bias assessment	11	Specify the methods used to assess risk of bias in the included studies, including details of the tool(s) used, how many reviewers assessed each study and whether they worked independently, and, if applicable, details of automation tools used in the process.	Appendix D
Effect measures	12	Specify for each outcome the effect measure(s) (e.g., risk ratio, mean difference) used in the synthesis or presentation of results.	page 4–6
Synthesis methods	13a	Describe the processes used to decide which studies were eligible for each synthesis (e.g., tabulating the study intervention characteristics and comparing against the planned groups for each synthesis (item 5)).	page 2
	13b	Describe any methods required to prepare the data for presentation or synthesis, such as handling of missing summary statistics, or data conversions.	page 2
	13c	Describe any methods used to tabulate or visually display results of individual studies and syntheses.	page 3
	13d	Describe any methods used to synthesize results and provide a rationale for the choice(s). If meta-analysis was performed, describe the model(s), method(s) to identify the presence and extent of statistical heterogeneity, and software package(s) used.	page 3
	13e	Describe any methods used to explore possible causes of heterogeneity among study results (e.g., subgroup analysis, meta-regression).	page 3
	13f	Describe any sensitivity analyses conducted to assess robustness of the synthesized results.	page 3
Reporting bias assessment	14	Describe any methods used to assess risk of bias due to missing results in a synthesis (arising from reporting biases).	page 3
Certainty assessment	15	Describe any methods used to assess certainty (or confidence) in the body of evidence for an outcome.	Appendix E
RESULTS			
Study selection	16a	Describe the results of the search and selection process, from the number of records identified in the search to the number of studies included in the review, ideally using a flow diagram.	Figure 1
	16b	Cite studies that might appear to meet the inclusion criteria, but which were excluded, and explain why they were excluded.	Figure 1
Study characteristics	17	Cite each included study and present its characteristics.	Table 1
Risk of bias in studies	18	Present assessments of risk of bias for each included study.	Appendix D
Results of individual studies	19	For all outcomes, present, for each study: (a) summary statistics for each group (where appropriate) and (b) an effect estimate and its precision (e.g., confidence/credible interval), ideally using structured tables or plots.	page 4,6,7
Results of syntheses	20a	For each synthesis, briefly summarise the characteristics and risk of bias among contributing studies.	page 7
	20b	Present results of all statistical syntheses conducted. If meta-analysis was performed, present for each the summary estimate and its precision (e.g., confidence/credible interval) and measures of statistical heterogeneity. If comparing groups, describe the direction of the effect.	page 4,6,7
	20c	Present results of all investigations of possible causes of heterogeneity among study results.	page 3
	20d	Present results of all sensitivity analyses conducted to assess the robustness of the synthesized results.	Appendix G
Reporting biases	21	Present assessments of risk of bias due to missing results (arising from reporting biases) for each synthesis assessed.	page 4
Certainty of evidence	22	Present assessments of certainty (or confidence) in the body of evidence for each outcome assessed.	page 4,6,7
**DISCUSSION**			
Discussion	23a	Provide a general interpretation of the results in the context of other evidence.	page 9–10
	23b	Discuss any limitations of the evidence included in the review.	page 10
	23c	Discuss any limitations of the review processes used.	page 10
	23d	Discuss implications of the results for practice, policy, and future research.	page 10
**OTHER INFORMATION**			
Registration and protocol	24a	Provide registration information for the review, including register name and registration number, or state that the review was not registered.	page 2
	24b	Indicate where the review protocol can be accessed, or state that a protocol was not prepared.	page 2
	24c	Describe and explain any amendments to information provided at registration or in the protocol.	page 2
Support	25	Describe sources of financial or non-financial support for the review, and the role of the funders or sponsors in the review.	page 11
Competing interests	26	Declare any competing interests of review authors.	page 11
Availability of data, code and other materials	27	Report which of the following are publicly available and where they can be found: template data collection forms; data extracted from included studies; data used for all analyses; analytic code; any other materials used in the review.	page 11

**Table A2:** PRISMA—abstract checklist.

Topic	No.	Item	Reported?
**TITLE**			
**Title**	1	Identify the report as a systematic review.	Yes
**BACKGROUND**			
**Objectives**	2	Provide an explicit statement of the main objective(s) or question(s) the review addresses.	Yes
**METHODS**			
**Eligibility criteria**	3	Specify the inclusion and exclusion criteria for the review.	No
**Information sources**	4	Specify the information sources (e.g., databases, registers) used to identify studies and the date when each was last searched.	Yes
**Risk of bias**	5	Specify the methods used to assess risk of bias in the included studies.	Yes
**Synthesis of results**	6	Specify the methods used to present and synthesize results.	No
**RESULTS**			
**Included studies**	7	Give the total number of included studies and participants and summarise relevant characteristics of studies.	Yes
**Synthesis of results**	8	Present results for main outcomes, preferably indicating the number of included studies and participants for each. If meta-analysis was performed, report the summary estimate and confidence/credible interval. If comparing groups, indicate the direction of the effect (i.e., which group is favoured).	Yes
**DISCUSSION**			
**Limitations of evidence**	9	Provide a brief summary of the limitations of the evidence included in the review (e.g., study risk of bias, inconsistency and imprecision).	No
**Interpretation**	10	Provide a general interpretation of the results and important implications.	Yes
**OTHER**			
**Funding**	11	Specify the primary source of funding for the review.	Yes
**Registration**	12	Provide the register name and registration number.	Yes

## Appendix B.

**Table A3:** **MOOSE checklist**.

Item No.	Recommendation	Reported on Page No.
**Reporting of background should include**		
1	Problem definition	1–2
2	Hypothesis statement	2
3	Description of study outcome(s)	4,6–7
4	Type of exposure or intervention used	2
5	Type of study designs used	2
6	Study population	2
**Reporting of search strategy should include**		
7	Qualifications of searchers (e.g., librarians and investigators)	2
8	Search strategy, including time period included in the synthesis and keywords	2
9	Effort to include all available studies, including contact with authors	2
10	Databases and registries searched	2
11	Search software used, name and version, including special features used (e.g., explosion)	Manual
12	Use of hand searching (e.g., reference lists of obtained articles)	Appendix C
13	List of citations located and those excluded, including justification	Figure 1
14	Method of addressing articles published in languages other than English	2
15	Method of handling abstracts and unpublished studies	2
16	Description of any contact with authors	2
**Reporting of methods should include**		
17	Description of relevance or appropriateness of studies assembled for assessing the hypothesis to be tested	2
18	Rationale for the selection and coding of data (e.g., sound clinical principles or convenience)	2
19	Documentation of how data were classified and coded (e.g., multiple raters, blinding and interrater reliability)	4,6
20	Assessment of confounding (e.g., comparability of cases and controls in studies where appropriate)	Table 1
21	Assessment of study quality, including blinding of quality assessors, stratification or regression on possible predictors of study results	Appendix E
22	Assessment of heterogeneity	3
23	Description of statistical methods (e.g., complete description of fixed or random effects models, justification of whether the chosen models account for predictors of study results, dose-response models, or cumulative meta-analysis) in sufficient detail to be replicated	2
24	Provision of appropriate tables and graphics	Appendices

## Appendix C. Search Strategy

**Table A4:** **Searching details in different database**.

Database	Search Detail
PubMed	(“endoscope s”[All Fields] OR “endoscoped”[All Fields] OR “endoscopes”[MeSH Terms] OR “endoscopes”[All Fields] OR “endoscope”[All Fields] OR “endoscopical”[All Fields] OR “endoscopically”[All Fields] OR “endoscopy”[MeSH Terms] OR “endoscopy”[All Fields] OR “endoscopic”[All Fields]) AND (“combinable”[All Fields] OR “combinated”[All Fields] OR “combination”[All Fields] OR “combinational”[All Fields] OR “combinations”[All Fields] OR “combinative”[All Fields] OR “combine”[All Fields] OR “combined”[All Fields] OR “combines”[All Fields] OR “combining”[All Fields]) AND (“intrarenal”[All Fields] OR “intrarenally”[All Fields]) AND (“surgery”[MeSH Subheading] OR “surgery”[All Fields] OR “surgical procedures, operative”[MeSH Terms] OR (“surgical”[All Fields] AND “procedures”[All Fields] AND “operative”[All Fields]) OR “operative surgical procedures”[All Fields] OR “general surgery”[MeSH Terms] OR (“general”[All Fields] AND “surgery”[All Fields]) OR “general surgery”[All Fields] OR “surgery s”[All Fields] OR “surgerys”[All Fields] OR “surgeries”[All Fields]) AND (“nephrolithotomies”[All Fields] OR “nephrolithotomy”[All Fields]) AND (“percutaneous”[All Fields] OR “percutaneously”[All Fields] OR “percutanous”[All Fields]) **Translations** **endoscopic**: “endoscope’s”[All Fields] OR “endoscoped”[All Fields] OR “endoscopes”[MeSH Terms] OR “endoscopes”[All Fields] OR “endoscope”[All Fields] OR “endoscopical”[All Fields] OR “endoscopically”[All Fields] OR “endoscopy”[MeSH Terms] OR “endoscopy”[All Fields] OR “endoscopic”[All Fields] **combined**: “combinable”[All Fields] OR “combinated”[All Fields] OR “combination”[All Fields] OR “combinational”[All Fields] OR “combinations”[All Fields] OR “combinative”[All Fields] OR “combine”[All Fields] OR “combined”[All Fields] OR “combines”[All Fields] OR “combining”[All Fields] **intrarenal**: “intrarenal”[All Fields] OR “intrarenally”[All Fields] **surgery**: “surgery”[Subheading] OR “surgery”[All Fields] OR “surgical procedures, operative”[MeSH Terms] OR (“surgical”[All Fields] AND “procedures”[All Fields] AND “operative”[All Fields]) OR “operative surgical procedures”[All Fields] OR “general surgery”[MeSH Terms] OR (“general”[All Fields] AND “surgery”[All Fields]) OR “general surgery”[All Fields] OR “surgery’s”[All Fields] OR “surgerys”[All Fields] OR “surgeries”[All Fields] **percutaneous**: “percutaneous”[All Fields] OR “percutaneously”[All Fields] OR “percutanous”[All Fields] **nephrolithotomy**: “nephrolithotomies”[All Fields] OR “nephrolithotomy”[All Fields]
Cochrane	(percutaneous):ti,ab,kw AND (nephrolithotomy):ti,ab,kw AND (endoscopic):ti,ab,kw AND (intrarenal):ti,ab,kw AND (surgery):ti,ab,kw (Word variations have been searched)
Embase	(endoscopic AND combined AND intrarenal AND (‘surgery’/exp OR surgery)) AND (percutaneous AND (‘nephrolithotomy’/exp OR nephrolithotomy))

## Appendix D.

**Table A5:** Risk of bias in included studies.

First Author, Year	Random Sequence Generation (Selection Bias)	Allocation Concealment (Selection Bias)	Blinding of Participants and Personnel (Performance Bias)	Blinding of Outcome Assessment (Detection Bias)	Incomplete Outcome Data (Attrition Bias)	Selective Reporting (Reporting Bias)	Other Bias
Wen, 2016	Low	High	High	Unclear	Low	Low	Unclear

**Table A6:** Newcastle–Ottawa Scale quality assessment scale for cohort studies.

Author, Year	Representativeness of the Exposed Cohort	Selection of the Nonexposed Cohort	Ascertainment of Exposure	Demonstration that Outcome of Interest Was Not Present at Start of Study	Comparability of Cohorts on the Basis of the Design or Analysis	Assessment of Outcome	Was Follow-up Long Enough for Outcomes to Occur	Adequacy of Follow up of Cohorts	Total Score
Zhao, 2020	✵	✵	✵	✵	✵✵	✵	✵	✵	9
Hamamoto, 2014	✵	✵	✵	✵	✵✵	✵	✵	✵	9
Nuño, 2013	✵	✵	✵	✵	✵✵	✵		✵	8
Isac, 2013	✵	✵	✵	✵	✵✵	✵			7
Leng, 2018	✵	✵	✵	✵	✵✵	✵	✵	✵	9
Xu, 2019	✵	✵	✵	✵	✵✵	✵		✵	8

A study can be awarded a maximum of one star for each numbered item within the Selection and Exposure categories. A maximum of two stars can be given for Comparability

## Appendix E.

**Table A7:** GRADE Approach for Rating the Quality of Treatment Effect Estimate.

Certainty Assessment							No. of Patients		Effect		Certainty	Importance
№ of Studies	Study Design	Risk of Bias	Inconsist-ency	Indirect-ness	Impreci-sion	Other Considerations	Endoscopic Combined Intrarenal Surgery	Percutaneous Nephrolithotomy	Relative (95% CI)	Absolute (95% CI)		
**Initial stone free**												
7	observational studies and randomized control trial	not serious	not serious	not serious	not serious	strong association	337/401 (84.0%)	320/521 (61.4%)	**OR 3.50** (2.16 to 5.67)	**234 more per 1000** (from 161 more to 286 more)	⨁⨁⨁◯ MODERATE	CRITICAL
**Final stone free**												
5	observational studies and randomized control trial	not serious	not serious	not serious	not serious	strong association	247/271 (91.1%)	263/350 (75.1%)	**OR 3.06** (1.57 to 5.95)	**151 more per 1000** (from 75 more to 196 more)	⨁⨁⨁◯ MODERATE	CRITICAL
**Overall complications**												
7	observational studies and randomized control trial	not serious	not serious	not serious	not serious	strong association	65/401 (16.2%)	142/521 (27.3%)	**OR 0.45** (0.29 to 0.70)	**128 fewer per 1000** (from 175 fewer to 65 fewer)	⨁⨁⨁◯ MODERATE	CRITICAL
**Severe complications**												
6	observational studies and randomized control trial	not serious	not serious	not serious	not serious	strong association	16/328 (4.9%)	61/423 (14.4%)	**OR 0.29** (0.16 to 0.52)	**98 fewer per 1000** (from 118 fewer to 64 fewer)	⨁⨁⨁◯ MODERATE	CRITICAL
**Post-operative fever**												
5	observational studies and randomized control trial	not serious	not serious	not serious	serious ^a^	none	24/265 (9.1%)	29/327 (8.9%)	**OR 0.65** (0.34 to 1.24)	**29 fewer per 1000** (from 57 fewer to 19 more)	⨁◯◯◯ VERY LOW	CRITICAL
**Hemoglobin drop**												
5	observational studies	not serious	serious ^b^	not serious	serious ^a^	none	295	389	-	MD **0.8 g/dL lower** (1.64 lower to 0.04 higher)	⨁◯◯◯ VERY LOW	IMPORTANT
**Required blood transfusion**												
6	observational studies and randomized control trial	not serious	not serious	not serious	serious ^b^	strong association	4/328 (1.2%)	19/423 (4.5%)	**OR 0.33** (0.12 to 0.91)	**30 fewer per 1000** (from 39 fewer to 4 fewer)	⨁⨁◯◯ LOW	CRITICAL
**Operative time**												
6	observational studies and randomized control trial	not serious	serious ^b^	not serious	serious ^a^	none	328	423	-	MD **6.73 h lower** (19.91 lower to 6.46 higher)	⨁◯◯◯ VERY LOW	IMPORTANT
**Hospital stay**												
6	observational studies and randomized control trial	not serious	serious ^b^	not serious	serious ^a^	none	338	425	-	MD **2.05 days lower** (4.14 lower to 0.05 higher)	⨁◯◯◯ VERY LOW	IMPORTANT

CI: Confidence interval; OR: Odds ratio; MD: Mean difference. Certainty are rated with 4 grades (◯◯◯◯):very low (⨁◯◯◯), low (⨁⨁◯◯), moderate (⨁⨁⨁◯), high (⨁⨁⨁⨁). a: The number of patients is small, below the optimal information size; b: There was important heterogeneity. Overall, the point estimates are sparsely distributed, and the 95% CI only occasionally overlap; c: The effect was large (RR either >2.0 or <0.5 based on consistent evidence from at least 2 studies, with no plausible confounders); therefore, we upgrade the quality of evidence for this outcome by 1 level.

## Appendix F. Subgroup Analyses for Operation Type

A priori subgroup analysis was planned to explore the influence of miniaturized access or conventional access of the operation on the pooled effect estimates.

## Appendix H. Sensitivity Analyses

We assessed the robustness of treatment effects on outcomes via a sensitivity analysis based on excluding high risk of bias cohort studies.

## Appendix K.

**Table A8:** Summary of the Result of Meta-Regression Analysis Regarding Outcome Measurements and Publication Date.

Outcomes	Variables	Study (N)	Coefficient (95% CI)	*p*-Value
Initial stone free rate	Publication date	7	−0.124 (−0.278 to 0.030)	0.1156
Final stone free rate		5	−0.200 (−0.412 to 0.013)	0.0654
Overall complications		7	−0.093 (−0.244 to 0.058)	0.2276
Severe complications		6	0.052 (−0.188 to 0.292)	0.6704
Postoperative fever		5	0.067 (−0.320 to 0.454)	0.7357
Hemoglobin drop		5	0.886 (0.697 to 1.127)	0.3254
Required blood transfusion		6	−0.020 (−0.407 to 0.366)	0.9187
Operative time		6	0.124 (0.000 to 67.032)	0.5154
Hospital stay		6	1.034 (0.449 to 2.381)	0.9373

## Appendix L.

**Table A9:** Summary of the Result of Meta-Regression Analysis Regarding Outcome Measurements and Amplatz Sheath Size.

Outcomes	Variables	Study (N)	Coefficient (95% CI)	*p*-Value
Initial stone free rate	Amplatz sheath size	7	0.034 (−0.078 to 0.146)	0.5501
Final stone free rate		5	0.058 (−0.121 to 0.238)	0.5227
Overall complications		7	0.054 (−0.019 to 0.126)	0.1468
Severe complications		6	−0.006 (−0.150 to 0.139)	0.9399
Postoperative fever		5	0.529 (−0.109 to 1.167)	0.1043
Hemoglobin drop		5	1.086 (0.894 to 1.320)	0.4036
Required blood transfusion		6	0.044 (−0.174 to 0.263)	0.6900
Operative time		6	9.308 (0.621 to 139.593)	0.1064
Hospital stay		6	1.405 (0.801 to 2.465)	0.2362
